# The use of incisional vacuum-assisted closure system following one-stage incision suture combined with continuous irrigation to treat early deep surgical site infection after posterior lumbar fusion with instrumentation

**DOI:** 10.1186/s13018-021-02588-y

**Published:** 2021-07-09

**Authors:** Hang Shi, Lei Zhu, Zan-Li Jiang, Zhi-Hao Huang, Xiao-Tao Wu

**Affiliations:** Department of Spine Surgery, Zhongda Hospital, School of Medicine, Southeast University, Nanjing, 210009 Jiangsu China

**Keywords:** Vacuum-assisted closure, One-stage incision suture, Continuous irrigation, Early deep surgical site infection, Posterior lumbar fusion with instrumentation

## Abstract

**Background:**

Previous reports concerning deep surgical site infection (SSI) after posterior spinal instrumentation treated with vacuum-assisted closure (VAC) system indicated that most patients must suffer from a delayed incision suture. To date, there are no published reports about the application of incisional VAC following a one-stage incision suture in the treatment of spinal infections. The purpose of this study was to evaluate the feasibility and efficacy of using an incisional VAC system following a one-stage incision suture combined with continuous irrigation to treat early deep SSI after posterior lumbar fusion with instrumentation.

**Methods:**

Twenty-one patients who were identified as early deep SSI after posterior lumbar fusion with instrumentation were treated by incisional VAC following a one-stage incision suture combined with continuous irrigation at our spine surgery center between January 2014 and March 2020. Detailed data from medical records were collected and analyzed, including age, gender, primary diagnosis, original operation, number of VAC dressing changes, duration of continuous irrigation, hospital stay, risk factors for infection, bacteria type, and laboratory data. Clinical efficacy was assessed using the pre- and postoperative visual analog scale (VAS) for back pain and Kirkaldy-Willis functional criteria by regular follow-up.

**Results:**

All the patients were cured and retained implants with an average of 1.9 times of VAC dressing replacement, and an average of 10.2 days of continuous irrigation. There were significant differences between pre-operation and post-operation in ESR, CRP, and VAS score of back pain, respectively (P < 0.05). The satisfactory rate was 90.5% according to Kirkaldy-Willis functional criteria. One patient developed a back skin rash with itching around the wound because of long-time contact with the VAC dressing. There was no recurrent infection or other complications during follow-up.

**Conclusions:**

Our preliminary results support that the treatment protocol is feasible and effective to treat early deep SSI following posterior lumbar fusion with instrumentation.

## Introduction

Postoperative surgical site infection (SSI) is a common and potentially devastating complication following spinal surgery. It is associated with prolonged hospitalization, increased morbidity, excess expenditure, and poor outcomes [1]. SSI is divided into superficial, deep, or organ space infection according to the United States Center for Disease Control and Prevention [2]. Deep SSI after posterior spinal instrumentation presents a treatment dilemma for surgeons in terms of how to completely eliminate bacteria and retain spinal instrumentation [3].

The treatments of deep SSI after posterior spinal instrumentation are varied and controversial, including sensitive antibiotics alone, surgical debridement, continuous suction irrigation, vacuum-assisted closure (VAC) system, hyperbaric oxygen therapy, temporary implantation of antibiotic cement, local tissue flap coverage, and removal of instrumentation if necessary [3–6]. Among them, the application of negative pressure wound therapy through VAC system has been the increasing focus on better management of deep spinal wound infections [7, 8]. However, previous reports concerning deep SSI after posterior spinal instrumentation treated with a VAC system indicated that most patients must suffer from delayed wound closure, regardless of whether they required multiple VAC replacements [4, 8–10]. Currently, some scholars have tried to use the incisional VAC system as a prophylactic treatment to prevent SSI in spinal surgery and concluded that it can significantly reduce the incidence of SSI [11, 12].

To date, there are no published reports about the application of incisional VAC following a one-stage incision suture in the treatment of spinal infections. Mediouni et al. [13, 14] proposed the concept of a “translational orthopaedist” who can move an idea all the way from basic research to clinical application and supported the application of a “T-model” that aims to build a greater interconnection between basic sciences and clinical sciences. Based on the above theory, in this study, we used an incisional VAC system following a one-stage incision suture combined with continuous irrigation to treat early deep SSI after posterior lumbar fusion with instrumentation and to evaluate the feasibility and efficacy of this treatment method.

## Materials and methods

### Patient population

This was a retrospective study. The study was performed in compliance with ethical standards and was approved by the institutional review board of our hospital. Informed consent documents were obtained from all patients prior to surgical treatments. The data were prospectively collected in 21 consecutive patients who were identified as early deep SSI after posterior lumbar fusion with instrumentation, treated by incisional VAC following a one-stage incision suture combined with continuous irrigation at our spine surgery center between January 2014 and March 2020. The cases consisted of 20 patients who underwent initial surgical treatments at our hospital and 1 patient who had received the initial surgery at another hospital. The initial diagnoses included lumbar isthmic spondylolisthesis, lumbar degenerative spondylolisthesis, lumbar disc herniation, and lumbar spinal stenosis. Patients with superficial SSI, late deep SSI, or those younger than 18 years of age were excluded from the study.

### Identification and diagnosis of early deep SSI

The definition of SSI was based on the guidelines from the United States Centers for Disease Control and Prevention [2, 15]. Deep SSI was defined as SSI that involved subfascial tissue [15]. However, the diagnosis criteria of early deep SSI were not uniform among the previous studies [9, 16–18]. In this study, the early deep SSI was specified as acute spinal infection occurring within 30 days after spinal surgery and involving subfascial soft tissue along with at least one of the following criteria: purulent drainage from deep tissues; a wound that dehisced or was deliberately opened by a surgeon and was cultured as positive or not cultured when redness, swelling, fever, pain, or tenderness to palpation was present; abscess harvested from revision surgery; other evidence of infection confirmed by histopathologic or radiologic examinations; and a diagnosis of a deep SSI by a surgeon [12, 15].

### Treatment protocol

**Fig. 1:**
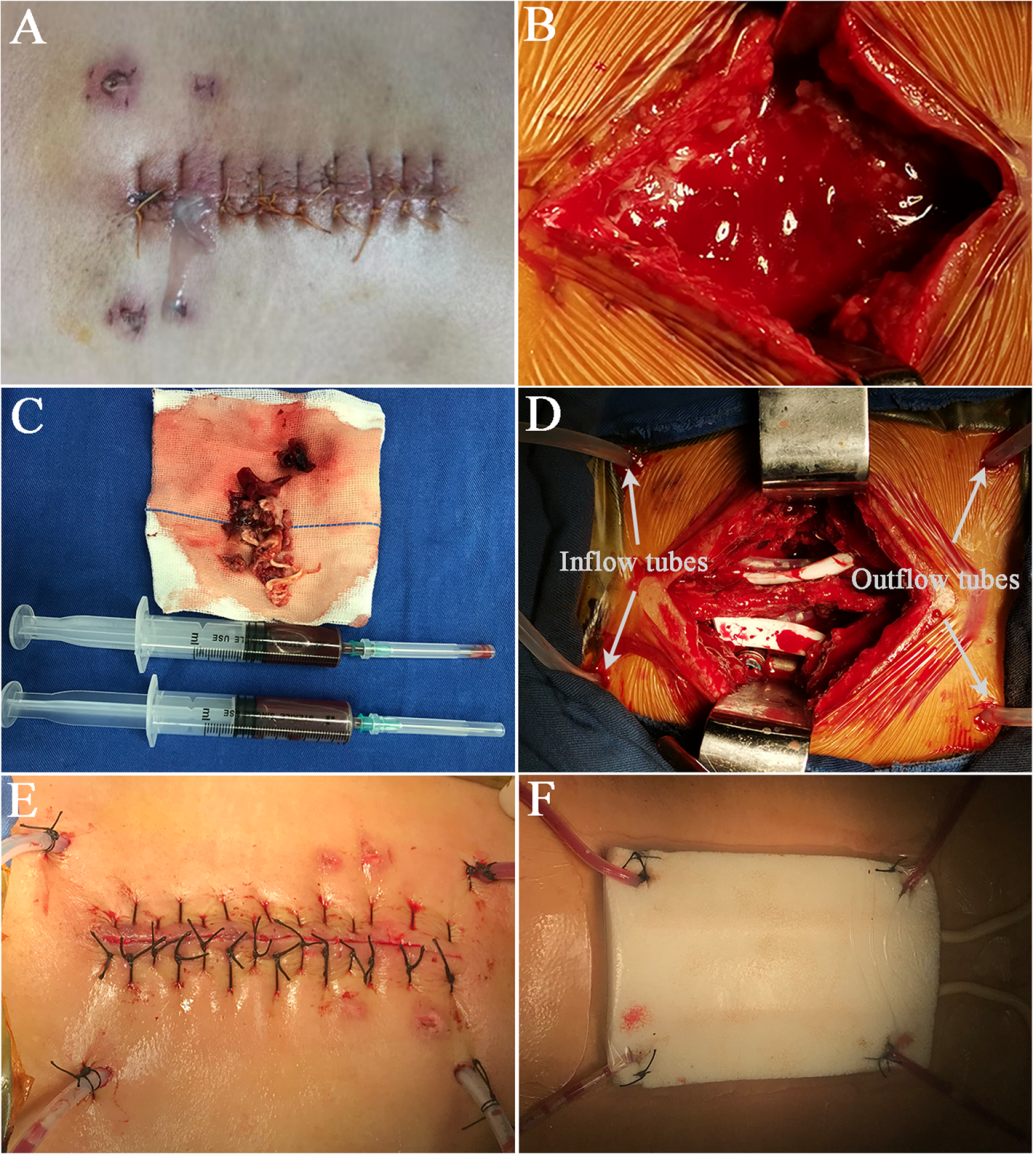
Diagnosis of early deep SSI and application of incisional VAC foam dressing. **A** The purulent drainage from a surgical site was found on the 10th day after L4-5 transforaminal lumbar interbody fusion, which was cultured with methicillin-sensitive *Staphylococcus aureus* infection. **B** The surgical site was adequately exposed. **C** The infected tissues and pus were sent to microbiology for bacterial culture and antibiotic susceptibility testing. **D** Two inflow tubes were placed under the deep fascia for continuous irrigation, and the other two outflow tubes were placed for drainage. **E** The infected wound after debridement was sutured for one stage. **F** An incisional VAC foam dressing was applied to fully cover the wound and the area where inflow and outflow tubes come out of the skin, followed by placement of an occlusive transparent film over the incision and surrounding wound area to make an airtight wound seal

After the diagnosis of early deep SSI (Fig. 1A), patients were taken to the operation room and underwent meticulous surgical procedures. All the procedures were performed under general anesthesia by the same surgical team. During the operation, the surgical site was adequately exposed (Fig. 1B) and then the infected tissues and pus were sent to microbiology for bacterial culture and antibiotic susceptibility testing (Fig. 1C). After a complete removal of infected and necrotic tissues or bone grafts, the wound was repeatedly irrigated with large amounts of hydrogen peroxide, normal saline, and iodine volt solution. After thorough debridement and irrigation with implant retention, one or two inflow tubes were placed under the deep fascia for continuous irrigation, and one or two outflow tubes depending on whether the spinous process was retained during the primary operation were placed for drainage (Fig. 1D). The irrigation solution was given with 80mg gentamicin per 500ml of normal saline. The fascia, subcutaneous tissues, and skin were closed routinely. After a one-stage incision suture (Fig. 1E), an incisional VAC foam dressing was applied to fully cover the wound and the area where inflow and outflow tubes come out of the skin, followed by placement of an occlusive transparent film over the incision and surrounding wound area to make an airtight wound seal (Fig. 1F). The suction tube attached to the VAC foam dressing was then connected to a suction device with a continuous negative pressure of 75 mmHg, the same as that applied by Dyck et al. [12], in order to pull the exudate into the VAC foam dressing and the container.

**Fig. 2:**
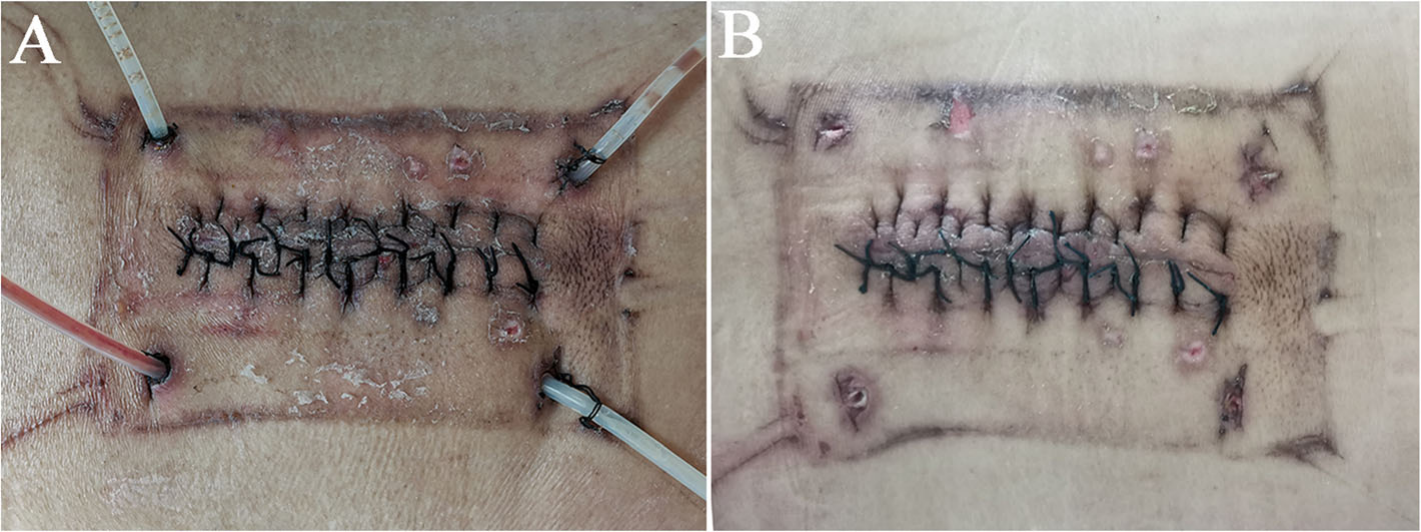
Management of VAC system and tubes. **A** The VAC system was removed, the continuous irrigation was terminated, and the inflow tubes were transformed into drainage tubes. **B** All the drainage tubes were removed, and the wound healed well

In the operation described above, the inflow velocity of solution for continuous irrigation was set at a minimum of 150 ml per hour on the first day after the operation and then was adjusted to 100 ml per hour for the next 2 days and to 50 ml per hour for the following 2 days. When the drainage was clear and the bacterial culture of the drainage was negative for three consecutive times, the VAC system was removed, the continuous irrigation was terminated, and the inflow tubes were transformed into drainage tubes (Fig. 2A). When the 24-h drainage volume was less than 50 ml, all the tubes were removed (Fig. 2B). In addition to that, the VAC dressing was changed every 5 to 7 days on the ward.

Intravenous broad-spectrum antibiotics were administered as soon as an early deep SSI was suspected. Then, they were replaced by sensitive antibiotics based on the results of postoperative bacterial culture and antibiotic susceptibility testing for 2 to 4 weeks and eventually followed by 6 weeks of oral antibiotics under the guidance of an infectious disease specialist.

### Data collection and efficacy assessment

Detailed demographic characteristics and medical records were collected and analyzed, including age, gender, primary diagnosis, original operation, number of VAC dressing changes, duration of continuous irrigation, hospital stay, risk factors for infection, bacteria type, and laboratory data. All patients were followed up regularly at 3, 6, and 12 months postoperatively and then annually. Laboratory data containing erythrocyte sedimentation rate (ESR) and C-reactive protein (CRP) level were examined periodically. Clinical efficacy was assessed using the pre- and postoperative visual analog scale (VAS) for back pain and Kirkaldy-Willis functional criteria [19]. During the follow-up period, all patients were monitored for recurrent infection or other complications.

### Statistical analysis

The statistical analysis was performed using SPSS Statistics software (version 22.0, SPSS Inc, Chicago, IL, USA). The values of VAS, ESR, and CRP were summarized using mean ± standard deviation (SD). The differences of the above continuous variables before and after surgery were analyzed using Dunnett t test, with *P* < 0.05 considered to be statistically significant.

## Results

Twenty-one patients in this study all received follow-up for an average of 19.2 months (range 13–38 months). All patients underwent a posterior lumbar fusion with instrumentation. The mean age of 21 patients, including 12 males and 9 females, at surgery was 62.9 years (range 34–79 years). The mean hospital stay was 30.4 days (range 24–53 days). The mean number of VAC dressing changes was 1.9 times, with 1 time for 4 patients, 2 times for 16 patients, and 3 times for 1 patient. The mean duration of continuous irrigation was 10.2 days (range 5–16 days) (Table 1).

**Table 1:** Patient and treatment characteristics

ID	Age	Sex	Primary diagnosis	Original operation	Number of VAC dressing change	Irrigation duration (days)	Hospital stay (days)
1	57	F	LSS	L4-S1 TLIF	1	7	29
2	73	M	LSS, LDH	L3-5 TLIF	2	10	24
3	71	M	LSS	L4-5 TLIF	2	11	27
4	59	M	LSS	L4-S1 TLIF	2	10	26
5	58	F	LIS, LDH	L4-S1 TLIF	2	11	37
6	46	F	LSS	L4-5 TLIF	1	6	29
7	74	M	LSS, LDH	L1-5 TLIF	2	10	33
8	34	F	LIS	L4-5 TLIF	2	12	28
9	79	M	LSS	L4-S1 PLIF	1	7	25
10	66	M	LSS, LDH	L3-5 TLIF	2	11	33
11	65	F	LDS, LSS	L4-S1 TLIF	3	16	53
12	68	M	LSS, LDH	L1-5 TLIF	2	9	34
13	48	M	LSS	L4-5 TLIF	2	13	31
14	77	F	LIS, LSS	L3-5 TLIF	2	8	32
15	53	M	LSS	L5-S1 TLIF	1	5	27
16	72	M	LIS	L4-5 PLIF	2	8	35
17	63	F	LIS	L4-5 TLIF	2	12	26
18	57	M	LSS, LDH	L3-5 TLIF	2	11	27
19	74	F	LSS	L3-5 TLIF	2	14	31
20	51	M	LSS, LDH	L2-3 TLIF	2	13	24
21	76	F	LSS	L3-S1 TLIF	2	10	28

*VAC* vacuum-assisted closure, *F* female, *M* male, *LIS* lumbar isthmic spondylolisthesis, *LDH* lumbar disc herniation, *LSS* lumbar spinal stenosis, *LDS* lumbar degenerative spondylolisthesis, *TLIF* transforaminal lumbar interbody fusion, *PLIF* posterior lumbar interbody fusion

In terms of risk factors for SSI after spine surgery according to previous reports [1, 3, 15]. Elderly (age > 70 years), body mass index > 30, smoking, diabetes mellitus, coronary artery disease, chronic obstructive pulmonary disease, anemia, low serum albumin, operation time > 3h, and perioperative blood loss > 500 ml were recorded in this study (Table 2). 85.7% (18 in 21) of the patients had at least one of the above risk factors.

**Table 2:** Risk factors of 21 patients for early deep surgical site infection (SSI) after posterior lumbar fusion with instrumentation

Risk factors	Number of patients	%
Elderly (age >70 years)	8	38.1
Body mass index > 30	4	19.0
Smoking	5	23.8
Diabetes mellitus	6	28.6
Coronary artery disease	3	14.3
Chronic obstructive pulmonary disease	4	19.0
Anemia	2	9.5
Low serum albumin	4	19.0
Operation time > 3h	7	33.3
Perioperative blood loss > 500 ml	8	38.1

Table 3 lists the pathogens cultured from intraoperative specimens. Bacterial cultures were positive in 14 of the 21 patients (66.7%), including 1 polymicrobial infection and 13 monomicrobial infections. A total of 7 strains of gram-positive pathogens were identified, including 2 strains of methicillin-sensitive *Staphylococcus aur*eus (MSSA), 4 strains of methicillin-resistant *Staphylococcus aureus* (MRSA), and 1 strain of *Staphylococcus epidermidis*. In addition, 8 strains of gram-negative pathogens were found, including 5 strains of *Escherichia coli* and one each strain of *Enterobacter cloacae*, *Acinetobacter baumanni*, and *Pseudomonas aeruginosa*.

**Table 3:** Pathogen cultures of 21 patients

Pathogens	Number of patients
Monomicrobial	13
Polymicrobial	1
Gram-positive	7
MSSA	2
MRSA	4
*Staphylococcus epidermidis*	1
Gram-negative	8
Escherichia coli	5
*Enterobacter cloacae*	1
*Acinetobacter baumanni*	1
*Pseudomonas aeruginosa*	1
No pathogen	7

*MSSA* methicillin-sensitive, *Staphylococcus aureus*, *MRSA* methicillin-resistant *Staphylococcus aureus*

**Fig. 3:**
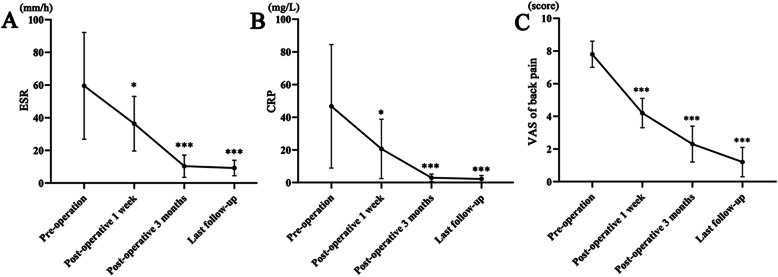
The values of ESR, CRP, and VAS score of back pain. **A** The ESR levels at pre-operation, 1 week, 3 months post-operatively, and the last follow-up. The ESR level at 1 week, 3 months post-operatively, and the last follow-up was significantly lower than that before the operation (*< 0.05, *** < 0.001). **B** The CRP levels at pre-operation, 1 week, 3 months post-operatively, and the last follow-up. The CRP level at 1 week, 3 months post-operatively, and the last follow-up was significantly lower than that before the operation (* < 0.05, *** < 0.001). **C** The VAS score of back pain at pre-operation, 1 week, 3 months post-operatively, and the last follow-up. The VAS score of back pain at 1 week, 3 months post-operatively, and the last follow-up was significantly lower than that before the operation (*** < 0.001)

In this study, the levels of ESR and CRP in each patient were higher than normal before debridement. The mean ESR level declined from 58.4 ± 32.3 pre-operatively to 34.1 ± 14.5, 10.6 ± 6.3 and 9.1 ± 4.2 at 1 week, 3 months post-operatively, and the last follow-up, respectively (Fig. 3A). The mean CRP level improved from 47.2 ± 38.4 pre-operatively to 21.5 ± 19.6, 2.9 ± 2.4 and 2.3 ± 2.0 at 1 week, 3 months post-operatively, and the last follow-up, respectively (Fig. 3B). The mean VAS score of back pain decreased from 7.6 ± 0.7 pre-operatively to 4.2 ± 0.8, 2.3 ± 1.0 and 1.2 ± 0.8 at 1 week, 3 months post-operatively, and the last follow-up, respectively (Fig. 3C). There were significant differences between pre-operation and post-operation in ESR, CRP, and VAS score of back pain, respectively (*P* < 0.05). ESR and CRP returned to normal levels within 3 months in most patients.

All patients were cured of the early deep SSI with implant retention. One patient developed a back skin rash with itching around the wound because of long time contact with the VAC dressing for 16 days, which was relieved after taking antiallergic drugs and removing the VAC dressing. There was no recurrent infection or other complications during follow-up. There were 7, 12, and 2 patients who had excellent, good, and fair outcomes, respectively, and no patients got poor outcomes according to Kirkaldy-Willis functional criteria^19^. The satisfactory rate was 90.5% (19/21).

## Discussion

Deep SSI after posterior spinal fusion with instrumentation is a troublesome and undesired complication, which may result in pseudarthrosis, spondylodiscitis, correction loss, spinal instability, adverse neurological sequelae, and implant removal if not treated timely [3]. Early diagnosis and aggressive surgical therapy are critical to eradicate infection, retain implant, decrease morbidity, restore spinal stability, and obtain satisfactory wound healing [8]. In terms of surgical treatments for early deep SSI after posterior spinal instrumentation, continuous irrigation suction system and VAC system following debridement are two common methods [4, 9, 20].

The application of a continuous irrigation suction system can improve local microcirculation, remove necrotic tissues and inflammatory factors, decrease the bacterial adhesion and recontamination, avoid secondary wound closure, and reduce the number of postoperative operating room visits. Lian et al. [20] reported that 23 patients with early postoperative deep SSI after spinal fusion with instrumentation were treated with thorough debridement and continuous irrigation suction system. The instrumentation and cages of all patients were retained successfully, and 21 patients (91.3%) had good wound healing with a mean of 12 days of continuous irrigation. Yuan et al. [9] reported 11 patients with early deep SSI after thoracolumbar instrumentation that underwent debridement and continuous irrigation suction system, and 3 patients required placement of the continuous irrigation suction system for a second time until wound healing. Rohmiller et al. [21] respectively studied 21 patients developing acute infections following posterior spinal fusion and instrumentation and found that 14 patients achieved complete resolution of their infection without recurrence after placement of the initial suction irrigation system during the follow-up period. However, there are some complications from a continuous irrigation suction system. First, the continuous irrigation suction system severely restricts the patient’s movement and functional exercise in bed. Besides, irrigation saline or drainage is likely to leak from inflow or outflow tubes, further resulting in wetting of the wound dressing, further increasing the frequency of dressing changes and the risk of retrograde infection.

The VAC system has been widely used to treat deep SSI after posterior spinal instrumentation because of its advantages of reducing tissue edema, promoting granulation tissue formation and angiogenesis, increasing blood flow, decreasing bacterial presence in the wound, and removing necrotic tissue [1, 4, 8, 9, 18]. Canavese et al. [22] reviewed 14 patients with early deep SSI after spinal instrumentation for scoliosis treated with a VAC system and identified that the VAC system was a reliable and useful tool for the spinal surgeon to eradicate the infections since all patients’ wounds healed with retention of the instrumentation and no loss of spinal correction or recurrent infection occurred. In the study performed by Zeng et al. [4], 16 patients with deep SSI after lumbar surgery with instrumentation underwent the treatment of the VAC system. The VAC dressing was replaced 2.4 times on average before secondary wound closure. All patients significantly improved the Japanese Orthopaedic Association scores and reduced the Oswestry disability index without recurrence of infection at the last follow-up. Nevertheless, the VAC system has some disadvantages. Jones et al. [23] ever reported five major complications related to the VAC system in four patients, including hemorrhage in two patients, one of whom died of unstable hemodynamics. Under some circumstances, granulation tissue may grow into the VAC dressing, thus affecting the efficacy of the VAC system. Webb [24] described that 2.2% of patients developed rash because of contact with the VAC dressing. Additionally, patients using the VAC system require a secondary closure, which adds to the patient’s pain and the extra cost.

In recent years, unlike many previous clinical applications of the VAC system for secondary wound closure, the incisional VAC system following primary wound closure has been successfully used for the prevention of SSI in spine surgery [11, 12]. However, there have been no reports about the application of incisional VAC system following a one-stage incision suture to treat deep spinal SSI. In this study, we treated 21 patients with early deep SSI after posterior lumbar fusion with instrumentation by using an incisional VAC system following a one-stage incision suture combined with continuous irrigation and obtained satisfactory results. To the best of our knowledge, this study is the first report to apply incisional VAC system following one-stage incision suture combined with continuous irrigation to the treatment of deep spinal SSI.

The treatment method we adopt in this study combined the advantages of a continuous irrigation suction system and VAC system and eliminated the disadvantages of each other. In our study, the incisional VAC system application can remove drainage and infectious material around the closure wound, prevent fluid leakage from inflow or outflow tubes, avoid skin erosion around inflow or outflow tubes, decrease the risk of retrograde infection, increase microcirculation and tissue regeneration, protect the closure wound from external infectious sources as a sterile barrier, decrease lateral tissue tension, and promote incisional apposition in case of dehiscence [25]. All the patients were cured and retained implants with an average of 1.9 times of VAC dressing replacement and an average of 10.2 days of continuous irrigation. No patients need to be transferred to the operation room for a second debridement. Only one patient developed a back skin rash with itching around the wound during treatment. There was no recurrent infection or other complications during follow-up. The postoperative VAS scores for back pain were significantly improved in all patients. The satisfactory rate was 90.5% according to Kirkaldy-Willis functional criteria [19].

With regard to laboratory testing related to SSI, ESR and CRP, as inflammatory markers, are two commonly used indicators to indicate improvement or progression of infection, especially CRP with more sensitivity [3, 26]. Under normal circumstances, peak ESR levels can be found up to 5–7 days and normalize within 4–6 weeks after surgery. CRP levels typically peak at 3 days and reduce within 10–14 days after surgery [3]. Therefore, the analyses of ESR and CRP levels must be interpreted in terms of the time since the index surgery. In the study by Yuan et al. [9], all the 23 patients with postoperative deep SSI showed increased CRP levels before debridement, and 21 patients (91.3%) presented with increased ESR levels. Zeng et al. [4] reported that all the 31 patients with postoperative deep SSI before debridement had elevated CRP levels, and 21 patients showed increased ESR levels. In our study, CRP and ESR levels increased in all patients with early deep SSI. Their levels dropped significantly after a series of aggressive treatments and returned to normal within 3 months in most patients.

Many scholars [3, 14, 26] have identified various risk factors for SSI after spine surgery, which can be classified as those intrinsic to patient-specific, procedure-related, and perioperative care. In this study, we took elderly (age > 70 years), body mass index > 30, smoking, diabetes mellitus, coronary artery disease, chronic obstructive pulmonary disease, anemia, low serum albumin, operation time > 3h, and perioperative blood loss > 500 ml as the risk factors for SSI and found that 18 patients (85.7%) had at least one of the above risk factors. In order to prevent postoperative SSI, we suggest as follows: First, surgeons should carefully consider patient’s potential risk factors for SSI and modify them as much as possible before surgery. Second, simplification of complex surgery and improvement of surgical technique are required to decrease operative time and intraoperative bleeding. Futhermore, postoperative careful incision care and timely correction of anemia or low serum albumin are also important to prevent complications of SSI.

We acknowledge there are some limitations to this study. First, the sample size of early deep SSI patients was relatively small. Second, there was no control group to further highlight the advantages of the treatment protocol in the study. Furthermore, this is a single-center retrospective study. To overcome these shortcomings, a multicenter prospective randomized controlled trial with a larger sample size should be performed to identify the efficacy of the treatment protocol used in this study.

## Conclusions

This study explores the feasibility and efficacy of an incisional VAC system following a one-stage incision suture combined with continuous irrigation for early deep SSI after posterior lumbar fusion with instrumentation. Our preliminary results support that the treatment protocol is feasible and effective to treat early deep SSI following posterior lumbar fusion with instrumentation. A multicenter prospective randomized controlled trial with a larger sample size should be conducted in the future.

## Data Availability

We state that the data will not be shared since all raw data used are presented in the figures or tables that are included in the article.

## Abbreviations

VAC: Vacuum-assisted closure
SSI: Surgical site infection
VAS: Visual analog scale
ESR: Erythrocyte sedimentation rate
CRP: C-reactive protein
MSSA: Methicillin sensitive *Staphylococcus aureus*
MRSA: Methicillin-resistant *Staphylococcus aureus*

## References

[1] Chen SH, Lee CH, Huang KC, Hsieh PH, Tsai SY (2015). Postoperative wound infection after posterior spinal instrumentation: analysis of long-term treatment outcomes. Eur Spine J..

[2] Edwards JR, Peterson KD, Mu Y, Banerjee S, Allen-Bridson K, Morrell G, Dudeck MA, Pollock DA, Horan TC (2009). National Healthcare Safety Network (NHSN) report: data summary for 2006 through 2008, issued December 2009. Am J Infect Control..

[3] Chen SH, Chen WJ, Wu MH, Liao JC, Fu CJ (2018). Postoperative infection in patients undergoing posterior lumbosacral spinal surgery: a pictorial guide for diagnosis and early treatment. Clin Spine Surg..

[4] Zeng J, Sun X, Sun Z, Guan J, Han C, Zhao X, Zhang P, Xie Y, Zhao J (2019). Negative pressure wound therapy versus closed suction irrigation system in the treatment of deep surgical site infection after lumbar surgery. World Neurosurg..

[5] Chen K, Lin JT, Sun SB, Lin J, Kong JZ, Tian NF (2018). Vacuum-assisted closure combined with a closed suction irrigation system for treating postoperative wound infections following posterior spinal internal fixation. J Orthop Surg Res..

[6] Laratta JL, Lombardi JM, Shillingford JN, Reddy HP, Gvozdyev BV, Kim YJ (2018). Permanent implantation of antibiotic cement over exposed instrumentation eradicates deep spinal infection. J Spine Surg..

[7] Daldal I, Senkoylu A (2020). Strategies of management of deep spinal infection: from irrigation and debridement to vacuum-assisted closure treatment. Ann Transl Med..

[8] Lee R, Beder D, Street J, Boyd M, Fisher C, Dvorak M, Paquette S, Kwon B (2018). The use of vacuum-assisted closure in spinal wound infections with or without exposed dura. Eur Spine J..

[9] Yuan W, Liu X, Zhou X (2018). Management of early deep wound infection after thoracolumbar instrumentation: continuous irrigation suction system versus vacuum-assisted closure system. Spine (Phila Pa 1976).

[10] Ousey KJ, Atkinson RA, Williamson JB, Lui S (2013). Negative pressure wound therapy (NPWT) for spinal wounds: a systematic review. Spine J..

[11] Adogwa O, Fatemi P, Perez E, Moreno J, Gazcon GC, Gokaslan ZL, Cheng J, Gottfried O, Bagley CA (2014). Negative pressure wound therapy reduces incidence of postoperative wound infection and dehiscence after long-segment thoracolumbar spinal fusion: a single institutional experience. Spine J..

[12] Dyck BA, Bailey CS, Steyn C, Petrakis J, Urquhart JC, Raj R, Rasoulinejad P (2019). Use of incisional vacuum-assisted closure in the prevention of postoperative infection in high-risk patients who underwent spine surgery: a proof-of-concept study. J Neurosurg Spine..

[13] Mediouni M, R Schlatterer D, Madry H, et al. A review of translational medicine. The future paradigm: how can we connect the orthopedic dots better? Curr Med Res Opin. 2018;34:1217-1229. 10.1080/03007995.2017.1385450 28952378

[14] Mediouni M (2019). A new generation of orthopaedic surgeons: “T-model”. Current Orthopaedic Practice..

[15] Mangram AJ, Horan TC, Pearson ML, Silver LC, Jarvis WR (1999). Guideline for prevention of surgical site infection, 1999. Centers for Disease Control and Prevention (CDC) Hospital Infection Control Practices Advisory Committee. Am J Infect Control..

[16] Liu JM, Deng HL, Chen XY (2018). Risk factors for surgical site infection after posterior lumbar spinal surgery. Spine (Phila Pa 1976).

[17] Canavese F, Krajbich JI (2010). Use of vacuum assisted closure in instrumented spinal deformities for children with postoperative deep infections. Indian J Orthop..

[18] Canavese F, Marengo L, Corradin M, Mansour M, Samba A, Andreacchio A, Rousset M, Dimeglio A (2018). Deep postoperative spine infection treated by negative pressure therapy in patients with progressive spinal deformities. Arch Orthop Trauma Surg..

[19] Kirkaldy-Willis WH, Farfan HF (1982). Instability of the lumbar spine. Clin Orthop Relat Res..

[20] Lian XF, Xu JG, Zeng BF, Liu XK, Li H, Qiu ML, Yang EZ (2014). Continuous irrigation and drainage for early postoperative deep wound infection after posterior instrumented spinal fusion. J Spinal Disord Tech..

[21] Rohmiller MT, Akbarnia BA, Raiszadeh K (2010). Closed suction irrigation for the treatment of postoperative wound infections following posterior spinal fusion and instrumentation. Spine (Phila Pa 1976).

[22] Canavese F, Gupta S, Krajbich JI (2008). Vacuum-assisted closure for deep infection after spinal instrumentation for scoliosis. J Bone Joint Surg Br..

[23] Jones GA, Butler J, Lieberman I, Schlenk R (2007). Negative-pressure wound therapy in the treatment of complex postoperative spinal wound infections: complications and lessons learned using vacuum-assisted closure. J Neurosurg Spine..

[24] Webb LX (2002). New techniques in wound management: vacuum-assisted wound closure. J Am Acad Orthop Surg..

[25] Horch RE (2015). Incisional negative pressure wound therapy for high-risk wounds. J Wound Care..

[26] Meredith DS, Kepler CK, Huang RC, Brause BD, Boachie-Adjei O (2012). Postoperative infections of the lumbar spine: presentation and management. Int Orthop..

